# Improved joint and patient-reported health assessments with pegloticase plus methotrexate co-therapy in patients with uncontrolled gout: 12-month exploratory outcomes of the MIRROR open-label trial

**DOI:** 10.1186/s13075-022-02979-4

**Published:** 2022-12-27

**Authors:** John K. Botson, Katie Obermeyer, Brian LaMoreaux, Lin Zhao, Michael E. Weinblatt, Jeff Peterson

**Affiliations:** 1 Orthopedic Physicians Alaska, 3801 Lake Otis Parkway, Anchorage, AK 99508 USA; 2 Horizon Therapeutics plc, Deerfield, IL USA; 3 Division of Rheumatology, Immunology and Immunity, Brigham and Women’s Hospital, Boston, MA USA; 4 Western Washington Arthritis Clinic, Bothell, WA USA

**Keywords:** Pegloticase, Uncontrolled gout, Refractory gout, Immunomodulation, Methotrexate

## Abstract

**Background:**

Uncontrolled/refractory gout patients are recalcitrant/intolerant to oral urate-lowering therapies (ULTs), experiencing frequent gout flares, functionally limiting tophi, and low quality of life. Pegloticase lowers urate, but anti-pegloticase antibodies limit urate-lowering efficacy and increase infusion reaction (IR) risk. Immunomodulator + pegloticase co-administration may improve treatment response rates, with 79% of MIRROR open-label trial (MIRROR-OL, pegloticase + oral methotrexate) participants meeting 6-month response criteria. Exploratory outcomes from MIRROR-OL are described here.

**Methods:**

Adults with uncontrolled gout (serum urate [SU] ≥ 6 mg/dL and ULT-intolerance/recalcitrance or functionally limiting tophi) were included. Oral methotrexate (15 mg/week) was administered 4 weeks before and during pegloticase treatment (biweekly 8 mg infusion, ≤ 52 weeks). Exploratory outcomes included change from baseline (CFB) in number of affected joints, Health Assessment Questionnaires (HAQs), and Gout Global Assessments.

**Results:**

Fourteen patients received ≥ 1 pegloticase infusion, with 13 included in 52-week analyses (1 enrolled before treatment-extension amendment, exited at 24 weeks). Three patients prematurely exited due to SU rise; 10 completed 52-week evaluations (8 completed 52 weeks of co-therapy, 2 completed 24 weeks [met treatment goals]). At 52 weeks, SU averaged 1.1 ± 2.5 mg/dL, with improvements in HAQ pain and health (CFB: − 33.6 and − 0.7, respectively), Patient and Physician Global Assessments (CFB: − 4.6 and − 5.7, respectively), and joint involvement (CFB: − 5.6, − 8.4, − 6.0 tender, swollen, tophi-affected joints, respectively). Two patients underwent dual-energy computed tomography, showing concomitant monosodium urate volume reductions. All patients had ≥ 1 AE, with 92.9% experiencing acute flare. One mild IR (“cough”) occurred and no new safety signals were identified.

**Conclusion:**

Pegloticase + methotrexate co-therapy resulted in sustained SU-lowering with meaningful improvements in clinical measures, urate burden, and patient-reported outcomes.

**Trial registration:**

ClinicalTrials.gov (NCT03635957)

## Introduction

Hyperuricemia and gout affect 32.5 million and 9.2 million people, respectively, in the USA [1]. Chronic hyperuricemia results in monosodium urate crystal deposition throughout the body, including in the kidneys, heart, larynx, and bowel [2], and is associated with a specific set of comorbidities, including cardiovascular disease [3–7], hypertension [7, 8], diabetes [7, 9], and chronic kidney disease [7, 10]. Even in patients with asymptomatic hyperuricemia, inflammation levels are elevated [11, 12]. When oral urate-lowering therapies (ULTs), including both xanthine oxidase inhibitors [13] and uricosurics [13, 14], are not tolerated, contraindicated, or no longer effective, refractory or uncontrolled gout can occur. The clinical hallmarks of uncontrolled gout include recurrent debilitating flares, chronic synovitis, chronic gout-related pain, tophi, urate-related joint damage (as visible on radiography), and urate deposition (as visible on dual-energy computed tomography [DECT] or ultrasound imaging). Uncontrolled gout further increases the risk for cardiovascular disease, diabetes, and chronic kidney disease [15] as well as the burden on quality of life resulting from ongoing pain and high levels of disability [16, 17]. Unfortunately, patients with chronic refractory gout are left with limited treatment options for urate lowering.

Pegloticase is an effective medication for treating uncontrolled gout, but some patients develop anti-drug antibodies (ADAs), which are associated with loss of urate-lowering efficacy [18–20] and increased infusion reactions (IRs) [18, 19]. In the phase 3 clinical trials, the pooled pegloticase responder rate at the approved dose regimen was 43.5% during month 6 (42% during months 3 and 6 combined [18]), with most high-titer ADA patients having loss of pegloticase efficacy within 6 months [18]. The 6-month results from the MIRROR open-label trial suggest that methotrexate (MTX) administered in conjunction with pegloticase nearly doubles the responder rate (79% during month 6 [11 of 14 patients], 95% confidence interval: 49–95%) with lower IR occurrence and otherwise similar safety profile as pegloticase monotherapy [21]. Furthermore, 12-month MIRROR open-label safety and efficacy findings indicate that urate-lowering is sustained over the long-term in patients remaining on therapy with both pegloticase and methotrexate [22]. Here, longer-term (12-month) exploratory endpoints of the MIRROR open-label trial are reported, including joint involvement, Health Assessment Questionnaire, and Global Assessments of Gout.

## Methods

This multi-center, open-label, single-arm efficacy and safety study (NCT03635957) was approved by the Western IRB (Puyallup, WA). Patients provided written informed consent and all study conduct adhered to the tenets of the Declaration of Helsinki.

### Study subjects

Adult patients with uncontrolled gout who were 65 years of age or younger were included. Uncontrolled gout was defined as serum urate (SU) ≥ 6 mg/dL with at least one of the following: SU ≥ 6 mg/dL despite oral ULT use, intolerance to ULT, or functionally limiting tophaceous deposits detected on clinical examination or with DECT. Key exclusion criteria included acute bacterial infection within 2 weeks of screening, active or recurrent bacterial infection, immunocompromised status, glucose-6-phosphate dehydrogenase (G6PD) deficiency, severe renal impairment (glomerular filtration rate [GFR] < 25 ml/min/1.73 m^2^ or currently on dialysis), alcohol consumption (> 3 drinks/week), liver disease (elevated [> 3 times upper limit of normal] alanine aminotransferase [ALT] or aspartate aminotransferase [AST]), and contraindication to MTX.

### Study medications

Study medications have been previously described [21, 22]. Briefly, this study included screening, a 4-week MTX run-in period (week − 4 through day 1), and a pegloticase + MTX co-therapy treatment period. Follow-up evaluations were performed 3 and 6 months after the end of the treatment period. Participants were administered gout flare prophylaxis at least 1 week prior to and during pegloticase treatment as recommended by the American College of Rheumatology [23]. Flare prophylaxis included colchicine, non-steroidal anti-inflammatory drugs (NSAIDs), and/or low-dose prednisone (≤ 10 mg/day) at the enrolling investigator’s discretion. Oral MTX (15 mg/week) and folic acid (1 mg/day) were initiated in all patients 4 weeks prior to the first pegloticase infusion (day 1) and were continued during the pegloticase + MTX (biweekly 8 mg pegloticase infusions) treatment period, which originally had a duration of 26 weeks but the study was extended to 52 weeks (protocol amendment). IR prophylaxis was administered prior to each pegloticase infusion (oral fexofenadine, oral acetaminophen, and IV methylprednisolone or hydrocortisone). To decrease IR risk, an established SU monitoring protocol [24] was followed, with patients discontinuing pegloticase (and MTX) if two consecutive SU measurements above 6 mg/dL were noted beginning at Week 2.

### Exploratory endpoints

The study’s primary endpoint (6-month pegloticase response) has been fully described elsewhere [21], as has efficacy, safety, pharmacokinetic, and pegloticase immunogenicity findings through 12 months [22]. The full study schedule of events is included in those publications. Exploratory outcomes reported here include mean change from baseline at weeks 14, 24, 36, and 52 for the number of tender (68 count) and swollen (66 count) joints, Health Assessment Questionnaire (HAQ) scoring, and Patient and Physician Global Assessments of Gout (GAs). The change from baseline in the number of joints affected by tophi was also evaluated at weeks 24, 36, and 52. HAQ pain and HAQ health both have a maximum score of 100 (pain: 0 [no pain] to 100 [severe pain], minimum clinically important difference [MCID] = 10 [25]; health: 0 [very well] to 100 [very poor]; MCID = 10) and the HAQ-Disability Index (HAQ-DI) had a maximum score of 3 (0 [no disability] to 3 [maximum disability], MCID for improvement = − 0.22 [26]). GAs were assessed using patient and physician responses to the following questions, respectively: “Considering all the ways that gout affects you, circle the number below that best represents how your gout has affected you over the last week” and “Considering the subject’s overall health related to gout, rate their gout overall.” Both patient and physician responded using a numeric rating scale for overall gout-related health ranging from 0 (excellent) to 10 (very poor], MCID = 1). The change in urate deposition volume, as measured on DECT imaging, was examined on available images.

### Statistical analyses

Statistical methods for the MIRROR open-label trial have been previously described [21]. Briefly, all exploratory analyses were performed on the modified intent-to-treat (mITT) population (all patients who received ≥ 1 pegloticase infusion) that remained in study past week 24. Some exploratory endpoints were also examined in patients who remained on therapy through week 52, including patient-reported outcomes (patient GA and HAQ measures) and joint involvement (number tender joints, number swollen joints, and number of tophi-affected joints). Baseline was defined as the last assessment prior to administration of the first pegloticase infusion.

Data are presented as mean ± standard deviation or *n* (%), as appropriate. Two-tailed paired *t* tests were performed to compare baseline and week 52 values.

## Results

A total of 14 patients with uncontrolled gout received at least one pegloticase infusion and made up the 6-month mITT population. Patients were all male and had an average age of 49.3 ± 8.7 years (Table 1). At baseline, mean SU was 9.2 ± 2.5 mg/dL, mean gout duration was 13.8 ± 7.4 years, and 13 patients (92.9%) had visible (subcutaneous) tophi. Twelve patients (85.7%) had previously used an oral ULT and were noted as refractory/intolerant. One patient who enrolled in the study prior to the treatment extension amendment exited the study per protocol at 24 weeks as a treatment responder. Therefore, 13 patients made up the mITT population for endpoint analyses after week 24. Three patients prematurely discontinued pegloticase + MTX due to loss of SU-lowering effect (2, 3, and 5 infusions received) and exited the study. Therefore, 10 patients were evaluated for the week 52 exploratory endpoints.

**Table 1:** Baseline characteristics of the modified intent-to-treat population

	(***N*** = 14)
Patient characteristics	
Age, mean ± SD, years	49.3 ± 8.66
Male sex, *n* (%)	14 (100)
Race, *n* (%)	
White	12 (85.7)
Asian	2 (14.3)
Body mass index (BMI), mean ± SD, kg/m^2^	33.9 ± 6.96
Smoking status, *n* (%)	
Never	4 (28.6)
Current	5 (35.7)
Former	5 (35.7)
Comorbidities, *n* (%)	
Vascular disorders	7 (50.0)
Hypertension	5 (35.7)
Deep vein thrombosis	1 (7.1)
Peripheral venous disease	1 (7.1)
Thrombosis	1 (7.1)
Varicose vein	1 (7.1)
Respiratory, thoracic, and mediastinal disorders^a^	6 (42.9)
Gastrointestinal disorders^b^	5 (35.7)
Nervous system disorders^c^	4 (28.6)
Psychiatric disorders^d^	4 (28.6)
Elevated alanine aminotransferase	3 (21.4)
Obesity	3 (21.4)
General disorders and administration site conditions^e^	3 (21.4)
Hepatobiliary disorders^f^	2 (14.3)
Nephrolithiasis	2 (14.3)
Erectile dysfunction	1 (7.1)
Atrial fibrillation	1 (7.1)
Gout characteristics and assessments	
Prior occurrence of tophi, *n* (%)	13 (92.9)
Time since first gout diagnosis, mean ± SD, years	13.8 ± 7.44
Serum uric acid, mean ± SD, mg/dL	9.2 ± 2.49
Prior oral urate-lowering therapy use	12 (85.7)
Allopurinol	11 (78.6)
Febuxostat	3 (21.4)
Probenecid	0

Baseline defined as the last assessment prior to the first pegloticase infusion

^a^Includes asthma, nasal congestion, deviated nasal septum, pulmonary mass, sleep apnea, and wheezing (*n* = 1 each)

^b^Includes alcoholic pancreatitis, dental caries, diarrhea, intestinal diverticulum, dyspepsia, gastrointestinal hemorrhage, and impacted tooth (*n* = 1 each)

^c^Includes headache (*n* = 2), cerebrovascular accident (*n* = 1), and paresthesia (*n* = 1)

^d^Includes anxiety, attention deficit/hyperactivity disorder, bipolar disorder, depression, abnormal orgasm, and insomnia (*n* = 1 each)

^e^Includes edema, peripheral edema, and ulcer (*n* = 1 each)

^f^Includes hepatic steatosis and NAFL (*n* = 1 each)

**Fig. 1:**
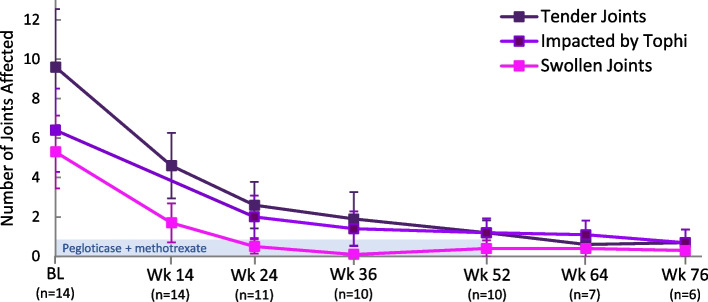
Summary of joint involvement in patients with uncontrolled gout treated with pegloticase (biweekly 8 mg infusion) plus oral methotrexate (15 mg/week) for up to 52 weeks (light blue bar; 52 weeks [26 infusions] in 8 patients, 24 weeks [12 infusions] in 2 patients). Data represent mean values and error bars represent standard error. Weeks 64 and 76 represent the 3- and 6-month post-treatment follow-up visits, respectively. BL, baseline (last measurement prior to the first pegloticase infusion). Reproduced from *Annals of the Rheumatic Diseases* 80 (Suppl 1) 2021 with permission from BMJ Publishing Group Ltd

As previously reported [22], SU rapidly declined with pegloticase initiation and, in responders remaining on treatment, was maintained well below 6 mg/dL throughout the 52-week pegloticase + MTX treatment period. In the 10 patients with SU measurements at week 52, mean SU was 1.1 ± 2.5 mg/dL (Table 2). In the 8 patients remaining on study treatment through week 52 (each received 26 pegloticase infusions), all 8 had sustained SU lowering, with undetectable SU levels through week 52. The remaining 2 patients met gout treatment goals at week 24, discontinuing pegloticase and MTX (12 pegloticase infusions administered) and beginning allopurinol at the treating investigators’ discretion (1 patient began allopurinol at week 24, 1 patient at week 26). With this sustained SU lowering, the mean number of tender, swollen, and tophi-affected joints progressively decreased in a clinically meaningful way throughout the treatment period (Table 2, Fig. 1), and, at end of treatment (6 months in 3 patients, 12 months in 8 patients), 10 patients (90.9%) had no detectable swollen joints and 7 patients (63.6%) had no tender joints. These improvements were maintained through the 6-month post-treatment evaluation. Of the 2 patients who discontinued pegloticase at week 24 and began allopurinol shortly after, TJC and SJC were unchanged between weeks 24 and 52.

**Table 2:** Summary of gout and clinical joint assessments in patients remaining in study through week 52 (*n* = 10)

	Baseline^**a**^	Week 52	Change from baseline	p-value
Serum uric acid, mean ± SD, mg/dL	9.3 ± 3.0	1.1 ± 2.5	− 8.2 ± 4.1	0.0001
Number of joints affected by gout, mean ± SD				
Swollen joints (66 count)	6.0 ± 8.0	0.4 ± 1.3	− 5.6 ± 7.7	0.048
Tender joints (68 count)	9.6 ± 11.9	1.2 ± 2.0	− 8.4 ± 10.8	0.036
Joints affected by tophi	7.2 ± 9.2	1.2 ± 2.3	− 6.0 ± 7.0	0.024
Health Assessment Questionnaire (HAQ), mean ± SD				
Disability index (MCID = − 0.22)	0.8 ± 0.9	0.1 ± 0.2	− 0.66 ± 0.88	0.041
HAQ pain score (MCID = 10)	39.8 ± 24.3	6.2 ± 8.6	− 33.6 ± 25.2	0.002
HAQ health score (MCID = 10)	41.2 ± 30.9	29.0 ± 39.4	− 12.2 ± 54.8	0.499
Global Assessments of Gout, mean ± SD				
Patient assessment (MCID = 1)	5.7 ± 2.0	1.1 ± 1.3	− 4.6 ± 2.1	< 0.0001
Physician assessment (MCID = 1)	6.0 ± 2.8	0.3 ± 0.5	− 5.7 ± 2.6	< 0.0001

Eight patients remained on pegloticase + methotrexate co-therapy thru week 52; 2 patients met treatment goals at week 24, discontinuing pegloticase + methotrexate co-therapy, remaining in study, and beginning allopurinol at the treating physician’s discretion

^a^Baseline value was the last measurement obtained prior to the first pegloticase infusion. HAQ Disability Index range: 0 to 3 (higher score indicates worse disability). HAQ pain range: 0 (no pain) to 100 (severe pain). HAQ health range: 0 (very well) to 100 (very poor). Global Assessment range: 0 (excellent) to 10 (very poor)

**Fig. 2:**
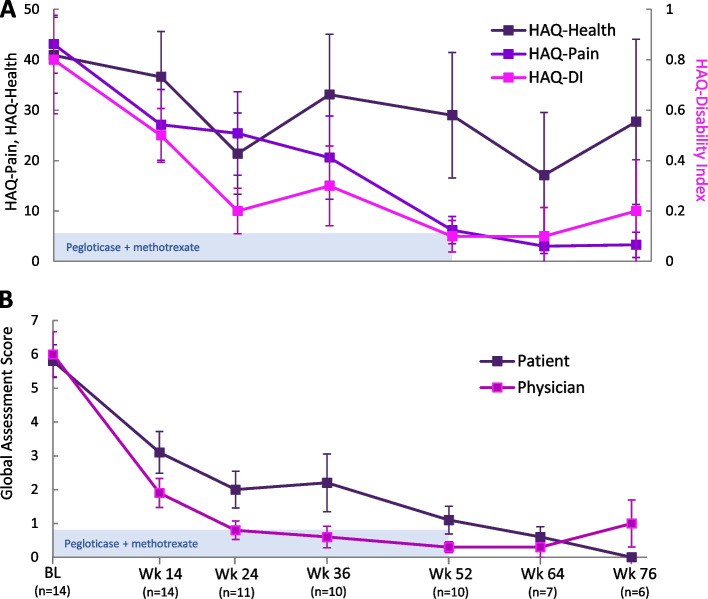
Summary of patient-reported HAQ outcomes during and after pegloticase + methotrexate treatment (light blue bar; 52 weeks in 8 patients, 24 weeks in 2 patients; **A**). Patient and physician Global Assessments of Gout are also shown (**B**). Data represent mean values and error bars represent standard error. Weeks 64 and 76 represent the 3- and 6-month post-treatment follow-up visits, respectively. Part A reproduced from *Annals of the Rheumatic Diseases* 80 (Suppl 1) 2021 with permission from BMJ Publishing Group Ltd

Progressive and clinically important improvements in HAQ pain, health, and DI scores were observed over the 52-week treatment period and were sustained during the 6-month follow-up period (Table 2, Fig. 2A). At week 52, HAQ pain, health, and DI scores were 6.2 ± 8.61, 29.0 ± 39.36, and 0.14 ± 0.199, respectively, all of which had improved from baseline (week 52 change from baseline—pain: − 33.6 ± 25.2, health: − 12.2 ± 54.8, DI: − 0.66 ± 0.88; *n* = 10). In the 2 patients who remained in study after stopping pegloticase at week 24 (re-initiating allopurinol), no clear trend in HAQ measures emerged after pegloticase discontinuation.

Both Patient and Physician Global Assessments of Gout also improved with study treatment (Table 2, Fig. 2B). At week 52, mean Patient Global Assessment score was 1.1 ± 1.29 (change from baseline: − 4.6 ± 2.07, *n* = 10) and mean Physician Global Assessment score was 0.3 ± 0.48 (change from baseline: − 5.7 ± 2.58, *n* = 10). Further, both Physician and Patient Global Assessment scores were 0 (“excellent health”) or 1 in the majority of subjects at week 52 (patient: 60%, physician: 90%). In the 2 patients who re-initiated allopurinol following 24 weeks of pegloticase therapy, patient GA worsened in one patient and improved in the other, and physician GA remained unchanged in both patients.

**Fig. 3:**
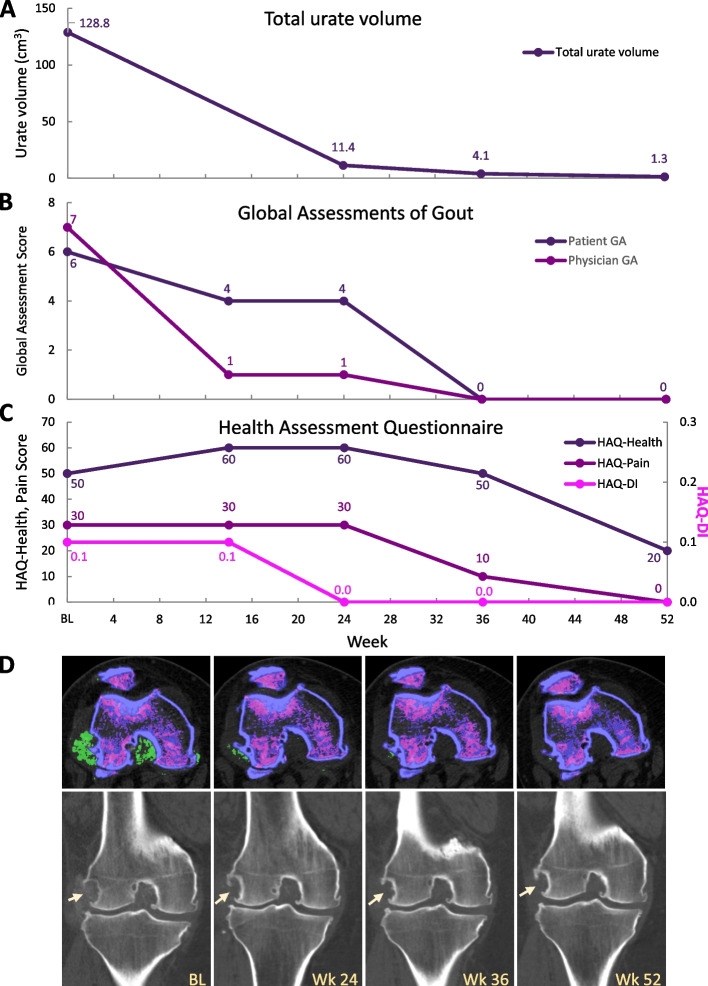
Clinical and patient-reported measures (**A**–**C**) and coincident DECT imaging (**D**) in a 44-year-old man with uncontrolled gout who underwent pegloticase + methotrexate co-therapy for 52 weeks. Marked improvements in quality of life measures were observed as urate load decreased. Left knee joint urate volume decreased from 22.6 cm^3^ at baseline to 3.1, 1.2, and 0.2 cm^3^ at weeks 24, 36, and 52, respectively (urate depicted in green). Lateral femoral condyle bone erosion (arrows) decreased in size and showed evidence of healing with increased sclerosis and new bone formation. DECT, dual-energy computed tomography; BL, baseline; HAQ, Health Assessment Questionnaire; DI, disability index. Part D reproduced from *Annals of the Rheumatic Diseases* 80 (Suppl 1) 2021 with permission from BMJ Publishing Group Ltd

Serial DECT images were available in 2 patients, with both having a marked, progressive decrease in urate deposition volume [27]. One patient received 26 infusions over 52 weeks and had a 99.0% reduction in total urate volume (baseline: 128.8 cm^3^, week 52: 1.3 cm^3^) with concomitant clinical improvement (Fig. 3A-C). The second patient only received 5 pegloticase infusions over 10 weeks, discontinuing therapy because of two consecutive SU values > 6 mg/dL per the monitoring protocol. However, this patient still experienced a 57.6% reduction in total urate volume (baseline: 59.2 cm^3^, week 10: 25.1 cm^3^). Further, both patients had imaging evidence of improvement in several bone erosions, including erosion size reduction, increased sclerosis, and new bone formation (Fig. 3D) [27].

Safety findings from this trial are reported in detail elsewhere [21, 22]. Briefly, all 14 patients (100%) experienced at least one adverse event (AE) during the pegloticase + MTX treatment period, with acute gout flare most-commonly reported (13 patients [92.9%]). Diarrhea, nasopharyngitis, upper respiratory infection, muscle strain, and arthralgia were each reported in 3 patients (21.4%) and sinusitis, hypertension, and increased liver function test values were each reported in 2 patients (14.3%). AEs of special interest included IR, anaphylaxis, and cardiovascular event. Of these, one mild IR (reported as “cough”) occurred in 1 patient (7.1%).

## Discussion

This 52-week open-label clinical trial further demonstrated longer-term efficacy of MTX + pegloticase co-therapy for treating uncontrolled gout [22]. With the addition of MTX, a higher proportion of patients achieved sustained SU lowering during pegloticase treatment and, in comparison to 6-month pegloticase monotherapy, a lower incidence of IRs and otherwise similar safety profile. The resultant sustained urate-lowering resulted in meaningful improvements in clinical and patient-reported outcomes. The number of joints affected by gout decreased, as indicated by a progressive and marked decreases in tophaceous, tender, and swollen joint counts over the 52-week treatment period. Sustained SU lowering also resulted in concomitant decreases in HAQ pain and HAQ-DI scores, indicating quality of life improvement. The phase 3 pegloticase clinical trials, which had a lower proportion of treatment responders at 42%, also showed significant improvements at 24 weeks in the number of tender joints (7.4 fewer joints), HAQ-DI score (− 0.22 points), and HAQ pain score (− 11 points) [18].

It is well known that maintaining SU below 6 mg/dL decreases gout sequelae, including gout flares [28, 29], tophi [29, 30], and joint damage [28], and lower SU levels have been shown to speed these improvements [28, 30]. Furthermore, a treat-to-target approach with ULTs has been shown to decrease systemic inflammation, even during asymptomatic, intercritical periods [12]. The current study further demonstrated the clinical benefits and quality of life improvement associated with rapid and sustained urate lowering. In the two patients with available DECT imaging, clinical and patient quality of life improvements coincided with a decrease in total urate volume. One patient who underwent 52 weeks of pegloticase + methotrexate co-therapy had a 99% reduction in total urate volume. The other patient, who received 5 pegloticase infusions before discontinuing due to a rise in SU, had a 58% reduction in total urate volume after only 10 weeks of co-therapy. These images allowed direct visualization of deposited urate reduction and further support the findings of Mandell et al. [31], who reported rapid tophi shrinkage with pegloticase-induced SU lowering.

This trial was limited by its open-label design, absence of a control group, and small study population. However, a randomized, controlled trial directly comparing pegloticase + MTX to pegloticase + placebo (monotherapy) has recently completed (MIRROR RCT, NCT03994731). In summary, the primary, secondary, and exploratory endpoints of the MIRROR open-label trial together demonstrate an increased response rate to pegloticase + MTX co-therapy (79% during month 6 [21]) compared to that observed in the pegloticase monotherapy clinical trials (44% during month 6 [18]) and show marked reductions in urate burden that coincided with meaningful clinical and quality of life improvements. Early and efficient identification of uncontrolled gout, along with effective treatment aimed at lowering SU, may limit not just gout flares and tophi persistence, but also the associated pain, disability, and joint damage associated with persistent hyperuricemia and urate deposition.

## Data Availability

Horizon is committed to responsibly sharing data from the clinical trials we sponsor. Access to anonymized, individual, and trial-level data (analysis data sets) may be granted to qualified researchers for independent scientific research, provided the trials are not part of an ongoing or planned regulatory submission (including clinical trial data for unlicensed products and indications). Data may be requested by submitting a research proposal and Statistical Analysis Plan and will be provided following review and approval of the plan and execution of a Data Sharing Agreement. For more information, or to submit a request, please submit to medicalinformation@horizontherapeutics.com.

## Abbreviations

MTX: Methotrexate
ULTs: Urate-lowering therapy
DECT: Dual-energy computed tomography
ADA: Anti-drug antibody
IR: Infusion reaction
SU: Serum urate
HAQ: Health Assessment Questionnaire
GA: Global Assessment of Gout
MCID: Minimum clinically important difference
DI: Disability index
mITT: Modified intent-to-treat
